# Armet, an aphid effector protein, induces pathogen resistance in plants by promoting the accumulation of salicylic acid

**DOI:** 10.1098/rstb.2018.0314

**Published:** 2019-01-14

**Authors:** Na Cui, Hong Lu, Tianzuo Wang, Wenhao Zhang, Le Kang, Feng Cui

**Affiliations:** 1 State Key Laboratory of Integrated Management of Pest Insects and Rodents, Institute of Zoology, Chinese Academy of Sciences, Beijing 100101, People's Republic of China; 2 State Key Laboratory of Vegetation and Environmental Change, Institute of Botany, Chinese Academy of Sciences, Beijing 100093, People's Republic of China; 3 University of Chinese Academy of Sciences, Beijing 100049, People's Republic of China

**Keywords:** insect effector, Armet, salicylic acid, pathogen resistance, SAMT, SABP2

## Abstract

Effector proteins present in aphid saliva are thought to modulate aphid–plant interactions. Armet, an effector protein, is found in the phloem sap of pea-aphid-infested plants and is indispensable for the survival of aphids on plants. However, its function in plants has not been investigated. Here, we explored the functions of Armet after delivery into plants. Examination of the transcriptomes of *Nicotiana benthamiana* and *Medicago truncatula* following transgenic expression of Armet or infiltration of the protein showed that Armet activated pathways associated with plant–pathogen interactions, mitogen-activated protein kinase and salicylic acid (SA). Armet induced a fourfold increase in SA accumulation by regulating the expression of *SAMT* and *SABP2*, two genes associated with SA metabolism, in Armet-infiltrated tobacco. The increase in SA enhanced the plants' resistance to bacterial pathogen *Pseudomonas syringae* but had no detectable adverse effects on aphid survival or reproduction. Similar molecular responses and a chlorosis phenotype were induced in tobacco by Armet from two aphid species but not by locust Armet, suggesting that the effector function of Armet may be specific for aphids. The results suggest that Armet causes plants to make a pathogen-resistance decision and reflect a novel tripartite insect–plant–pathogen interaction.

This article is part of the theme issue ‘Biotic signalling sheds light on smart pest management’.

## 1. Introduction

As a group of piercing/sucking insects, aphids probe only mesophyll cells and ingest the cell contents through their stylets. During this process, aphids secrete saliva to enable them to feed stably and efficiently [1]. Aphid saliva is believed to contain effector proteins that potentially have similar functions in plants as their counterparts in pathogenic bacteria, fungi, oomycetes and nematodes [2]. The immune reactions of plants to aphid feeding are similar to their reactions to fungal or bacterial pathogen infection in terms of the plant–pathogen interaction pathways involved [3,4]. Although many proteins have been identified in the saliva of various kinds of aphids [5–7], the functions of most of them as effectors in aphid host plants remain elusive.

Hormone signalling plays a key role in plant immunity. Salicylic acid (SA) and jasmonic acid (JA) are regarded as major defence hormones in plants [8]. JA and jasmonates are ubiquitous signals for tissue injury and for the subsequent activation of plant defense responses to many herbivorous insects. Constitutive activation of JA signalling in *Arabidopsis thaliana* enhanced plant resistance to *Myzus persicae* [9], and blocking JA signalling promoted population growth in *M. persicae* and *Brevicoryne brassicae* [10]. The role of SA in resistance to piercing/sucking insects is controversial. Intercellular application of SA stimulated the defence responses of wheat against Russian wheat aphid (*Diuraphis noxia*) [11]. However, overexpression of SA-related genes and mutation of these genes showed that SA signalling was not critical for controlling *M. persicae* infestation of *A. thaliana* [3]. SA accumulation, activation of SA-responsive gene expression, and inhibition of JA-responsive gene expression have frequently been observed in plants such as wheat, barley, tomato and *A. thaliana* after infestation with various species of aphids [12,13]. Nonetheless, the identity of the effector(s) in aphid saliva that stimulates plant SA signalling remains unknown.

In our previous work, we reported that Armet protein of the pea aphid (*Acyrthosiphon pisum*) (ApArmet) is secreted into the phloem sap of fava beans together with the watery saliva of the aphid during the feeding process. As an effector, ApArmet induced the expression of genes involved in the plant–pathogen interaction pathway after inoculation into *Nicotiana benthamiana* leaves [14]. However, the molecular mechanisms through which Armet acts as an effector during the regulation of aphid–plant interactions are unknown. In the present study, we investigated the immune responses of two plant species, *N. benthamiana* and *Medicago truncatula*, to Armet through extracellular application and intracellular expression. In addition to activating the plant–pathogen interaction and mitogen-activated protein kinases (MAPK) signalling pathways, Armet was found to increase SA accumulation and activate SA-responsive gene expression. The SA accumulation induced by Armet conferred resistance to bacterial pathogens but not to aphids.

## 2. Material and methods

### (a) Plants and aphids

*Medicago truncatula* R108 and A17 and *N. benthamiana* were used in the study. Germinated seeds of *N. benthamiana* and *M. truncatula* were transplanted to 8 cm × 8 cm or 12-cm-diameter plastic pots filled with a mixture of nutrient soil and vermiculite (1 : 1, v/v) and cultured at 21°C with a long-day photoperiod (16 L : 8D) at 70% relative humidity. Populations of *M. persicae* and *A. pisum* were raised on *A. thaliana* and *M. truncatula* A17, respectively, in a growth chamber with a 16-h light photoperiod at 23°C.

### (b) RNA extraction and real-time quantitative PCR

Total RNA was isolated from *N. benthamiana* and *M. truncatula* leaves and from *M. persicae* and *Locusta migratoria*, and then was reverse-transcribed to cDNA. Real-time quantitative PCR (qPCR) was used to quantify the transcript levels of various genes in *N. benthamiana* and *M. truncatula*. qPCR was performed in a Light Cycler 480 II instrument (Roche, Basel, Switzerland). Differences were analysed using one-way ANOVA for multiple comparisons or the *t*-test for pairwise comparisons in SPSS 17.0. Detailed procedures can be found in electronic supplementary material, Materials and Methods.

### (c) Generation of transgenic *N. benthamiana* and *M. truncatula* expressing *ApArmet*

To generate transgenic *N. benthamiana* for *ApArmet* transient expression, *ApArmet* (XM_001949506 in GenBank) lacking the sequence encoding the signal peptide was cloned in the pENTR/D-TOPO vector using the pENTR/D-TOPO Cloning Kit (Invitrogen, Carlsbad, CA, USA) and the primer pair ApArmet-F/ApArmer-R (electronic supplementary material, table S1); it was then recombined with the destination vector pFAST-G02 using LR Clonase Enzyme Mix (Invitrogen). The ApArmet transient expression assay was performed by infiltrating four-week-old *N. benthamiana* leaves with *Agrobacterium tumefaciens* strain GV3101 (OD_600 nm_ = 1) carrying the recombinant destination vector. Leaves were collected at 66 h post-inoculation (hpi), and the transcript level of *ApArmet* was measured by qPCR. The pFAST-G02 empty vector was transformed as a negative control.

For the constitutive expression of *ApArmet* in *M. truncatula* R108, *ApArmet* lacking the sequence encoding the signal peptide was cloned in the pMDC32 Gateway vector with a 2 × 35S promoter using the primers ApArmet-KpnI-F/ApArmet-SpeI-R to generate the recombinant destination vector (electronic supplementary material, table S1). *Agrobacterium* transformation and the regeneration of *M. truncatula* R108 via somatic embryogenesis have been described previously [15]. For the detection of *ApArmet* in F_1_ plants, the *ApArmet* transgene was amplified by PCR using the primers *ApArmet-*q-F*/ApArmet-*q-R and genomic DNA that had been extracted from the leaves using the cetyltrimethylammonium ammonium bromide (CTAB) method. The expression level of *ApArmet* in positive transgenic F_1_ plants was measured by qPCR. The F_1_ plants with high *ApArmet* expression levels were self-fertilized. After determining the genotype separation ratio and the *ApArmet* expression level in the F_3_ generation, one line of the F_2_ generation of plants was selected for transcriptome sequencing. Native *M. truncatula* R108 was used as a negative control.

### (d) Protein expression, purification and infiltration of leaves

The cloning, expression and purification of ApArmet and the Armets from *M. persicae* (MpArmet) and *L. migratoria* (LmArmet) are described in electronic supplementary material, Materials and Methods. After dilution to a final concentration of 25 ng µl^−1^ or 50 ng µl^−1^ in buffer (20 mM Tris–HCl, 120 mM NaCl, pH 8.0), 100 µl purified ApArmet, MpArmet or LmArmet was infiltrated into four-week-old *N. benthamiana* leaves using a sterile 1-ml syringe and a 0.4 × 13 RWLB needle (Shanghai Misawa Medical Industry, Shanghai, China). The plants were photographed daily for 14 days. After concentration to 100 ng µl^−1^ using a Millipore ultrafiltration device, 5 µl of purified ApArmet protein was infiltrated into eight-week-old *M. truncatula* R108 leaves by microinjection through a glass needle at slow speed using a Nanoliter 2000 device (World Precision Instruments, Sarasota, FL, USA). An equal volume of purified product obtained from *Escherichia coli* transfected with the pET28a empty vector was infiltrated as a negative control. The leaves of *N. benthamiana* (25 ng µl^−1^ ApArmet), *M. truncatula* with ApArmet infiltration and the control group were collected at 60 hpi for transcriptome sequencing and qPCR.

### (e) Transcriptomic sequencing and analysis

Total RNAs were sent to the BGI Company (Shenzhen, China) for RNA-seq analysis using the single-end digital gene expression sequencing strategy. At least 12 million clean reads were obtained for each sample. Reads of each sample were deposited in the Short Read Archive of the National Center for Biotechnology Information (NCBI) under the accession number SRP149658. Differentially expressed genes were analysed with a fold change threshold ≥ 2 and divergence probability ≥ 0.8. Detailed procedures can be found in electronic supplementary material, Materials and Methods. (SAMT, salicylate carboxymethyltransferase; SABP2, salicylic acid-binding protein 2; SAGT, SA glucosyltransferase.)

### (f) Knockdown and overexpression of salicylate carboxymethyltransferase and salicylic acid binding protein 2 in tobacco leaves

The open reading frames (ORFs) of *SAMT* and *SABP2* were amplified from the cDNA of *N. benthamiana* using the primer pairs SAMT-F/SAMT-R and SABP2-F/SABP2-R, respectively, and inserted into the Gateway destination vector pEarleyGate100 using LR Clonase (Invitrogen) for overexpression (oeSAMT, oeSABP2; electronic supplementary material, table S1). An artificial microRNA corresponding to *SABP2* (amiRNA-*SABP2*) was designed and synthesized through several rounds of PCR using the primers SABP2-I miR-s, SABP2-II miR-a, SABP2-III miR*s, SABP2-IV miR*a, SABP2-A and SABP2-B (electronic supplementary material, table S1) based on the protocol described at http://wmd3.weigelworld.org and inserted into the pENTR/D-TOPO vector (Invitrogen). After sequence confirmation, amiRNA-*SABP2* was recombined into pEarleyGate100 using LR Clonase (Invitrogen) for SABP2-knockdown (miSABP2). The recombinant plasmids containing oeSAMT, oeSABP2 and miSABP2 were introduced into *N. benthamiana* leaves using *A. tumefaciens* strain GV3101 with the empty pEarleyGate100 vector as a negative control. Eighteen hours after introduction, the leaves were infiltrated with 2.5 µg purified ApArmet; the leaves were harvested 48 h later for measurement of gene expression and SA content. An equal volume of purified product obtained from the pET28a empty vector was infiltrated as a control. Six biological replicates with four leaves in each replicate were prepared.

### (g) Measurement of salicylic and jasmonic acids

The SA and JA content of ApArmet protein-infiltrated *N. benthamiana* leaves at 60 hpi was measured after grinding the leaves in liquid nitrogen. The SA content of ApArmet, oeSAMT, oeSABP2 and miSABP2 transgenic *N. benthamiana* leaves was measured at 66 hpi. SA and JA assays were conducted at the National Centre for Plant Gene Research (Beijing, China) using ultra-performance liquid chromatography-tandem mass spectrometry (UPLC-MS/MS) with single solid-phase extraction (SPE), purification and isotope dilution as previously described [16]. Three replicates were prepared for each group. Differences between the groups were statistically analysed using the independent-sample *t*-test in SPSS v. 17.0.

### (h) Survival and reproduction of aphids on ApArmet protein-infiltrated plants

After 24 h of infiltration of *N. benthamiana* and *M. truncatula* leaves with purified ApArmet, 10–15 first-instar nymphs of *M. persicae* and 15–18 third-instar nymphs of *A. pisum* were raised on the *N. benthamiana* and *M. truncatula* leaves, respectively, for 13 days. Leaves infiltrated with the purified product obtained from the pET28a empty vector were used as a negative control. Six biological replicates were prepared for each group. The survival rate and the number of offspring were recorded daily. The survival curves of the ApArmet protein-infiltrated and control groups were calculated using Kaplan–Meier method and statistical differences were analysed with the log-rank test in SPSS v. 17.0. The reproductive rate was expressed as the average number of offspring per aphid during the first 4 days of the oviposition period; differences were evaluated using the *t*-test in SPSS v. 17.0.

### (i) *Pseudomonas syringae* infection

*Pseudomonas syringae* pv. *tabaci* was cultured in King's B medium at 28°C for 24 h. After centrifugation at 4000 r.p.m. for 10 min, the bacterial cells were resuspended in 10 mM MgSO_4_ to a final density of 10^2^ colony-forming units (cfu)/ml. One day after ApArmet infiltration, one tobacco leaf was infiltrated with 100 µl of bacterial suspension at two sites. At 6 and 9 d post-infiltration (dpi) with bacteria, two 1-cm-diameter leaf discs covering the infiltration sites were excised, ground and suspended in 1 ml of 10 mM MgCl_2_, and 100 µl of the resulting suspension was plated on King's B agar plates and cultured for 24 h at 28°C before the measurement of bacterial cfu. Leaves infiltrated with purified product obtained from the pET28a empty vector were used as a control. Six biological replicates were prepared; the differences between the ApArmet-infiltration and control groups were evaluated using the *t*-test in SPSS v. 17.0.

### (j) Phylogenetic analysis

One hundred and thirty-three insect Armet homologous protein sequences were acquired via a BLASTP search of the non-redundant protein sequence database and a TBLASTN search of the expressed sequence tags in NCBI (http://www.ncbi.nlm.nih.gov/). The Armet sequences of *Hyalopterus persikonus*, *Sitobion avenae* and *Rhopalosiphum padi* were obtained from our previous transcriptomes [17,18]. Protein sequences were aligned using ClustalW, and an unrooted phylogenetic tree was constructed using the neighbour-joining method (*p*-distance and pairwise deletion) in MEGA 6.0. Bootstrap analysis of 1000 replicates was applied to evaluate the confidence level of the tree topology.

## 3. Results

### (a) Plant responses to Armet

**Figure 1.**
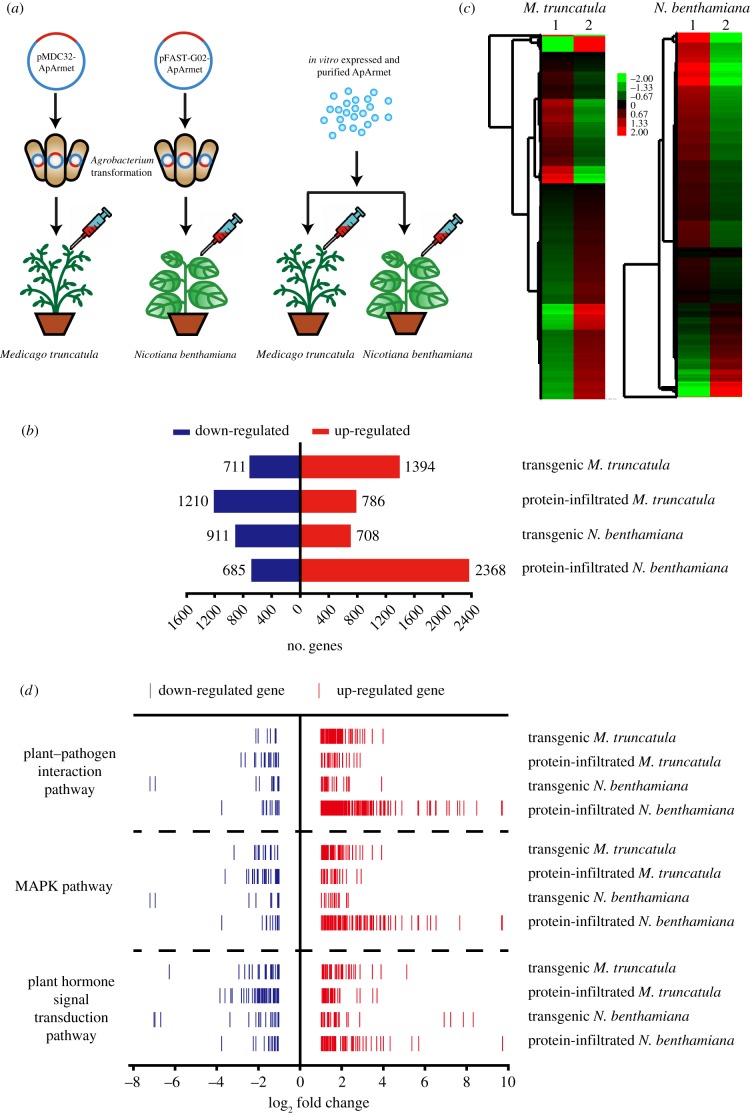
Transcriptomic analyses of *ApArmet* transgenic and protein-infiltrated *Medicago truncatula* and *Nicotiana benthamiana*. (*a*) Schematic showing the treatment of the two plants. (*b*) Number of upregulated and downregulated genes in the four groups of plants compared to the control groups. (*c*) Heat maps of the differentially expressed genes in the four groups of plants compared to the control groups. The heat maps show the fold change values on a log2 scale. Gene expression is shown as upregulated (red), downregulated (green) or no change (black). 1, ApArmet protein infiltration; 2, *ApArmet* transgenic expression. (*d*) Number and fold change in the expression of upregulated and downregulated genes in the plant–pathogen interaction, MAPK, and plant hormone signal transduction pathways. Each line represents a gene.

To investigate the function of Armet protein in plants, we generated transgenic *M. truncatula* that constitutively expressed *ApArmet* and transgenic *N. benthamiana* that transiently expressed *ApArmet*. *ApArmet* was expressed *in vitro*, purified and leaves of *M. truncatula* and *N. benthamiana* were infiltrated with 0.5 µg and 2.5 µg purified protein, respectively (figure 1*a*). The four groups of plants and their respective negative controls were subjected to transcriptomic analysis. In transgenic *M. truncatula* and *N. benthamiana*, 2105 and 1619 genes, respectively, were differentially expressed, whereas in protein-infiltrated *M. truncatula* and *N. benthamiana*, 1996 and 3053 genes, respectively, were differentially expressed (figure 1*b*). The overall variation in gene expression between the transgenic and protein-infiltrated plants was high (figure 1*c*). Only a small number of genes showed similar variation in expression in the transgenic and protein-infiltrated plants, i.e. there were 99 co-upregulated genes and 118 co-downregulated genes in *M. truncatula* and 165 co-upregulated genes and 93 co-downregulated genes in *N. benthamiana*.

The functional annotations for the differentially expressed genes included three KEGG pathways associated with pathogen or insect resistance in plants, i.e. the plant–pathogen interaction pathway, the MAPK signalling pathway and plant hormone signal transduction. The number and fold change in expression of the differentially expressed genes in these three KEGG pathways were comparable in the four groups of plants (figure 1*d*). Plant hormone signal transduction is one of the downstream components of the plant–pathogen interaction pathway and the MAPK signalling pathway. In *N. benthamiana* and *M. truncatula*, infiltration of ApArmet protein or transgenic expression of ApArmet activated the SA pathway by upregulating the expression of transcription factor TGA, pathogenesis-related protein 1 (PR1) and regulatory protein NPR1, and suppressed the JA pathway by upregulating the expression of JAZ protein, a transcriptional repressor of JA signalling.

### (b) Armet increases salicylic acid levels in plants

**Figure 2.**
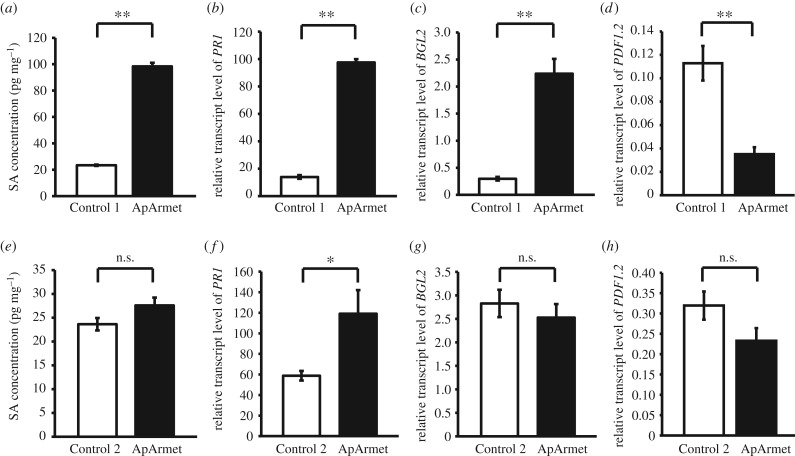
Effects of Armet on SA and JA signalling pathways in *Nicotiana benthamiana*. (*a*) SA concentration in *N. benthamiana* after ApArmet protein infiltration. (*b–d*) Relative transcript levels of the SA downstream genes *PR1* and *BGL2* and the JA downstream gene *PDF1.2* in *N. benthamiana* after ApArmet protein infiltration measured using real-time quantitative PCR (qPCR). (*e*) SA concentration in *ApArmet* transgenic *N. benthamiana*. (*f–h*) Relative transcript levels of *PR1*, *BGL2* and *PDF1.2* in *ApArmet* transgenic *N. benthamiana* measured using qPCR. The transcript level of each gene relative to that of *ef1α* is shown as the mean ± s.e. Control 1, plant infiltrated with the purified product from *E. coli* transfected with pET28a empty vector; Control 2, plant transformed with the pFAST-G02 empty vector. *p < 0.05; **p < 0.01; n.s., no significant difference.

After observing the effects of ApArmet on the expression of genes associated with the SA and JA pathways, we measured the concentrations of SA and JA in *N. benthamiana* using UPLC-MS/MS. The SA concentration in ApArmet protein-infiltrated tobacco plants was fourfold that found in control plants (figure 2*a*), whereas it did not change in the transgenic plants (figure 2*e*). We quantified the transcript levels of two SA-responsive genes, *PR1* and *beta-1,3-glucanase 2* (*BGL2*) [19] using qPCR. The transcript levels of *PR1* and *BGL2* in protein-infiltrated tobacco plants increased sixfold and 13-fold, respectively (figure 2*b,c*), whereas *PR1* expression was doubled in the transgenic tobacco (figure 2*f,g*). Although the amount of SA in the ApArmet protein-infiltrated tobacco plants increased, no change in the JA concentration in these plants was observed, probably because the basal level of JA in *N. benthamiana* is too low to be detected using our measurement platform (electronic supplementary material, figure S1). The transcript level of *defensin 1.2* (*PDF1.2*), a JA-responsive gene [8], was quantified and found to be downregulated significantly in the ApArmet protein-infiltrated tobacco plants and to remain unchanged in the transgenic tobacco plants (figure 2*d,h*). These results demonstrate that ApArmet stimulates SA signalling.

### (c) Armet regulates the expression of genes associated with salicylic acid metabolism

**Figure 3.**
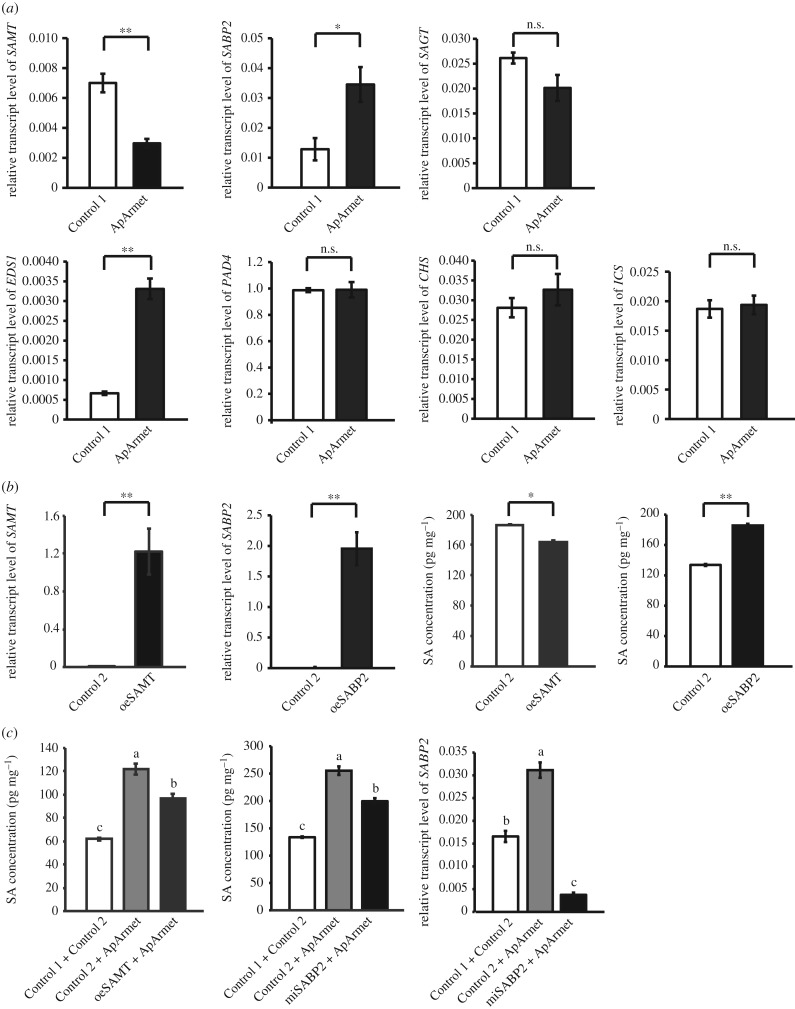
Armet regulates the expression of genes associated with SA metabolism. (*a*) The relative transcript levels of genes involved in the metabolism (*SAMT*, *SABP2* and *SAGT*), signal transduction (*EDS1* and *PAD4*) and biosynthesis (*ICS* and *CHS*) of the SA pathway in ApArmet protein-infiltrated *Nicotiana benthamiana*. (*b*) Relative transcript levels of *SAMT* and *SABP2* and SA concentrations in *SAMT*-overexpressing (oeSAMT) and *SABP2*-overexpressing (oeSABP2) transgenic *N. benthamiana*. (*c*) The SA concentrations in oeSAMT and *SABP2*-knockdown (miSABP2) transgenic *N. benthamiana* and the relative transcript level of *SABP2* in miSABP2 transgenic *N. benthamiana* after ApArmet protein infiltration. The transcript level of each gene relative to that of *ef1α* is shown as the mean ± s.e. Control 1, plant infiltrated with the purified product from the *E. coli* transfected with pET28a empty vector; Control 2, plant transformed with the pEarleyGate100 empty vector. *p < 0.05; **p < 0.01; n.s., no significant difference. Different lowercase letters indicate significant differences at the *p* < 0.05 level.

Why does Armet facilitate the accumulation of SA in plants? To answer this question, we measured the variation in the expression of genes involved in SA metabolism: *SAMT*, *SABP2* and *SA glucosyltransferase* (*SAGT*); SA signal transduction: *enhanced disease susceptibility 1 (EDS1)* and *phytoalexin deficient 4* (*PAD4*); and SA biosynthesis: *isochorismate synthase (ICS)* and *chalcone synthase (CHS)* [20] using qPCR in tobacco plants infiltrated with ApArmet protein. Only two metabolism genes, *SAMT* and *SABP2*, and one signal transduction gene, *EDS1*, showed significant changes in expression in the presence of Armet; Armet downregulated the expression of *SAMT* and upregulated the expression of *SABP2* and *EDS1* (figure 3*a*). The expression of two SA biosynthesis genes, *ICS* and *CHS*, did not change in the presence of Armet (figure 3*a*).

SAMT is a methyltransferase that converts SA to methyl salicylate, and SABP2 is a methyl esterase that converts methyl salicylate to SA [20]. To verify the roles of SAMT and SABP2 in the Armet-induced accumulation of SA, we transiently overexpressed *SAMT* (oeSAMT) and *SABP2* (oeSABP2), and knocked down *SABP2* expression (miSABP2) in tobacco plants. In the oeSAMT and oeSABP2 tobacco, the transcript levels of *SAMT* and *SABP2*, respectively, greatly increased (figure 3*b*). The content of SA was significantly reduced in the oeSAMT plants and significantly enhanced in the oeSABP2 plants (figure 3*b*), confirming the functions of these two proteins in the metabolism of SA in tobacco. In the miSABP2 tobacco plants, the transcript level of *SABP2* was reduced even in the presence of ApArmet (figure 3*c*). When ApArmet was infiltrated into transgenic oeSAMT or miSABP2 tobacco plants, SA accumulation was significantly lower than that in non-transgenic plants but still higher than that in control plants without ApArmet (figure 3*c*). These results indicate that the Armet-induced SA accumulation was due to the downregulation of *SAMT* and the upregulation of *SABP2*.

### (d) Armet enhances plant resistance to bacterial pathogen *P. syringae* but not to aphids

As a major defence hormone, SA may affect the fitness of insects or pathogens for growth on plants. Does the increased SA accumulation caused by Armet have effects on aphids or pathogens? We tested the performance of *M. persicae* on ApArmet protein-infiltrated tobacco leaves and that of *A. pisum* on ApArmet protein-infiltrated *M. truncatula* leaves. Neither the survival rate nor the reproductive rate of *M. persicae* and *A. pisum* changed when the aphids lived on Armet-infiltrated leaves rather than control leaves (electronic supplementary material, figure S2A-2D). At the same time, we measured the resistance of tobacco plants infiltrated with ApArmet to the bacterial pathogen *P. syringae* pv. *tabaci* by monitoring bacterial propagation in the leaves. The number of *P. syringae* pv. *tabaci* colonies obtained from ApArmet protein-infiltrated tobacco leaves was significantly reduced at 6 and 9 dpi (electronic supplementary material, figure S2E, 2F); it was only half of that obtained from the control group without ApArmet at 6 dpi. These results show that the increased level of SA produced after ApArmet infiltration enhances plant resistance to bacterial pathogens but not to aphids.

### (e) The effector functions of Armet are specific for aphid species

Armet is present in animal species ranging from invertebrates to mammals [14]. We compared the sequences of Armet from 133 insect species. Phylogenetic analysis showed that the Armet sequences from seven aphid species clustered together and that these sequences diverged greatly from the homologous sequences of other insect species (electronic supplementary material, figure S3A). The homology of the amino acid sequences of Armet from the seven aphid species was quite high, with identities greater than 95%. On the other hand, aphid Armet was largely differentiated from Armet of other insects, with which it displayed identities ranging from 50 to 60%. Compared to the carboxy-terminal region, the N-terminal portion of Armet was quite conserved within aphids but more divergent from that of other insects (electronic supplementary material, figure S3B).

**Figure 4.**
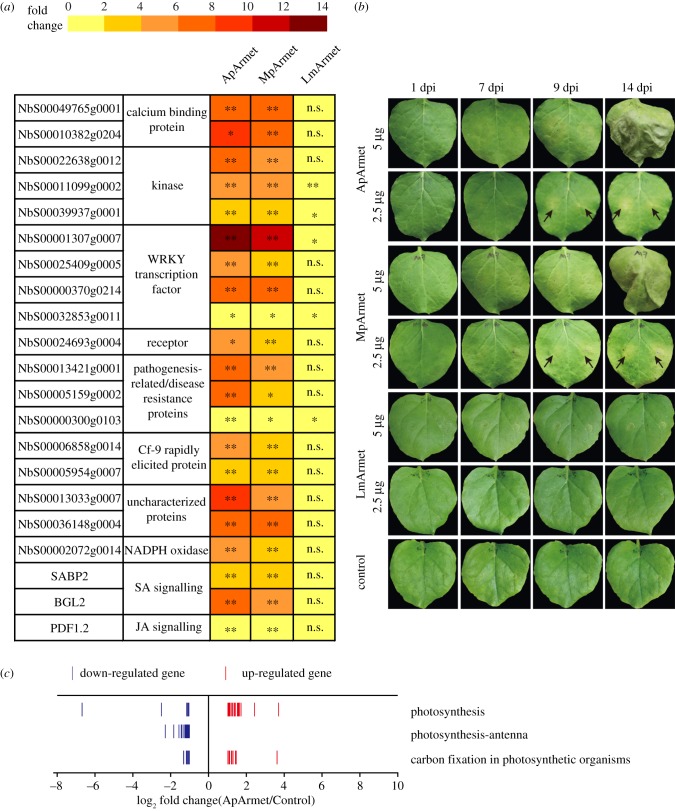
The effector functions of Armet are specific for aphid species. (*a*) Fold changes in the expression of genes of the plant–pathogen interaction pathway in *Nicotiana benthamiana* leaves infiltrated with aphid Armet (ApArmet and MpArmet) or locust Armet (LmArmet) and measured using real-time quantitative PCR. Leaves infiltrated with purified product from the pET28a empty vector were used as a control. *p < 0.05; **p < 0.01; n.s., no significant difference. (*b*) Chlorosis phenotype of *N. benthamiana* leaves at different days post-infiltration (dpi) of ApArmet, MpArmet or LmArmet protein. Leaves infiltrated with purified product from the pET28a empty vector were used as a control. The arrows indicate chlorosis. (*c*) Number and fold change in the expression of upregulated and downregulated genes in three photosynthesis-related pathways in the transcriptome of ApArmet protein-infiltrated *N. benthamiana*. Each line represents a gene.

To determine whether the functions of Armet are specific for aphid species, we cloned *Armet* sequences from the green peach aphid *M. persicae* (MpArmet) and the locust *Locusta migratoria* (LmArmet). After expression *in vitro*, purified MpArmet, LmArmet and ApArmet were infiltrated into tobacco *N. benthamiana* leaves. The expression levels of 18 genes in the plant–pathogen interaction pathway [14] and of *PR1*, *BGL2* and *PDF1.2* were quantified using qPCR and compared in protein-infiltrated and control tobacco plants. All 18 plant–pathogen interaction genes, as well as *PR1* and *BGL2*, were upregulated, and *PDF1.2* was downregulated by ApArmet or MpArmet, whereas only five plant–pathogen interaction genes were upregulated by LmArmet (figure 4*a*). Furthermore, *PR1*, *BGL2* and *PDF1.2* expression did not respond to LmArmet (figure 4*a*), indicating that, unlike aphid Armet, LmArmet did not influence SA or JA signalling transduction pathway.

In addition to measuring the gene expression response to the three Armet proteins, we also explored the phenotypes induced by these proteins in the leaves of tobacco *N. benthamiana*. A similar chlorosis phenotype of leaves was observed at 9 dpi after infiltration of the leaves with 2.5 µg ApArmet or MpArmet; when the amount of protein applied was increased to 5 µg, the leaves became wilted at 14 dpi (figure 4*b*). By contrast, no obvious symptoms appeared in leaves infiltrated with 2.5 or 5 µg of LmArmet or in control leaves (figure 4*b*). The chlorosis phenotype induced by ApArmet or MpArmet may be related to the negative effects of the proteins on the photosynthesis system. Examination of the transcriptome of ApArmet protein-infiltrated *N. benthamiana* showed that the expression of a large number of genes associated with the photosynthesis-antenna, photosynthesis and carbon fixation pathways (especially the photosynthesis-antenna pathway), was downregulated; 34 genes encoding chlorophyll a/b binding proteins were downregulated (figure 4*c*).

The results reported here show that the molecular response and the phenotype induced by the two aphid Armet proteins were similar, whereas locust Armet did not induce a strong response in plants. These findings indicate that the functions of Armet may be specific for aphid species.

## 4. Discussion

The immune responses of plants to aphid infestation are similar to the responses to pathogen infection, especially with respect to plant hormone signalling. The activation of plant SA signalling is the convergent point in the response to aphid infestation and pathogen infection. Prior to this study, the factors through which aphids induce SA signalling were unknown. The effects of SA signalling on aphids and other pathogens may differ. The accumulation of SA is detrimental to plant pathogens, but it is not clear that it is detrimental to aphid infestation. Our work identified the first known effector protein found in aphid saliva, Armet, that is capable of inducing an SA response to aphid feeding. Although Armet-induced SA accumulation was not harmful to aphid fitness, it conferred resistance to infection by the bacterial pathogen *P. syringae*, indicating that aphids deceive plants and cause them to make a pathogen-resistance decision.

Armet promotes the accumulation of SA, thereby increasing the resistance of plants to bacterial pathogens. JA is thought to be an efficient anti-insect hormone, whereas SA plays a key role in pathogen resistance and confers a less effective defence against insects than JA [21]. In most cases, the production of SA and JA is antagonistic [22]. Aphids use Armet to drive plant responses in the direction of an inefficient SA defence against aphids; this is reflected in the unimpaired survival rates and reproductive rates of *M. persicae* and *A. pisum* living on Armet-infiltrated leaves with elevated SA accumulation. Similar tripartite insect–plant–pathogen interactions have been observed. For example, feeding of the silverleaf whitefly *Bemisia argentifolii* on tomato significantly reduces the incidence of powdery mildew [23]. Infestation of rice by the white-backed planthopper *Sogatella furcifera* induces resistance to the rice blast fungus *Magnaporthe grisea* [24]. These resistance reactions are attributed to the accumulation of pathogenesis-related proteins or secondary metabolites induced by insect infestation.

Armet regulates the metabolism of SA via *SAMT* and *SABP2*. Armet does not influence the expression of SA synthesis genes; instead, it regulates the expression of *SAMT* and *SABP2* to promote SA accumulation. SAMT belongs to the SABATH family of methytransferases, which contains 24 members in *Arabidopsis* [25]. One homologous protein, NtSAMT of *Nicotiana tabacum*, converts SA to MeSA and has a high affinity for SA. Silencing of *NtSAMT* reduced MeSA levels in primary leaves and blocked systematic acquired resistance to tobacco mosaic virus [26]. The MeSA esterase activity of SABP2 (NtSABP2) has also been demonstrated in *N. tabacum* [27]. NtSAMT and NtSABP2 are the main enzymes that contribute to systemic acquired resistance in *N. tabacum*. *Arabidopsis* and *Solanum tuberosum* possess 18 and one orthologous SABP2 genes, respectively. Only one *SABP2* gene and 12 genes of the SABATH family were identified in the genome of *N. benthamiana*. In the present study, we cloned one *SAMT* gene and the unique *SABP2* gene of *N. benthamiana* and verified their functions in the metabolism of SA for the first time. Furthermore, the expression of these two genes was found to be regulated by aphid Armet. How aphid Armet regulates the expression of these two genes is a subject worthy of further research.

Aphid effectors play diverse roles in aphid–plant interactions. Armet and C002 from *A. pisum* are indispensable for normal feeding behaviour and survival of aphids on plants. Overexpression of C002 in plants enhanced aphid colonization, while infiltration of Armet protein did not affect aphid fitness on plants [28]. Two effectors (Me10 and Me23) of the potato aphid *Macrosiphum euphorbiae* enhanced aphid fecundity when delivered into *N. benthamiana* [29], whereas expression of the *M. persicae* effectors Mp10, Mp42, Mp56, Mp57 and Mp58 in *N. benthamiana* decreased aphid reproduction [30,31]. It is worth noting that Mp10 induced chlorosis and local cell death in tobacco from 2 dpi onward [32]. This chlorosis response was dependent on the ubiquitin ligase-associated protein SGT1, which is required for the activation of NBS-LRR proteins and plant resistance responses [33]. By contrast, Armet-induced chlorosis in tobacco appeared at 9 dpi, much later than Mp10-mediated chlorosis. We measured the SGT1 transcript level and found that the expression of this gene did not respond to Armet (data not shown). Armet-induced chlorosis may result from downregulation of the expression of chlorophyll a/b binding proteins, which are involved in the function of light-harvesting complexes I and II. The downregulation of chlorophyll a/b binding proteins decreases chlorophyll levels in *Arabidopsis* [34]. Therefore, the mechanisms underlying the chlorosis phenotype differ for Armet and Mp10.

In conclusion, aphids exploit Armet, a ubiquitous animal protein, as an effector to suppress the effective JA pathway through the modification of the SA pathway to benefit their feeding activity and increase plant resistance to bacterial pathogens. This function is likely specific to aphids. These results reflect an adaptation strategy used by aphids to exploit tripartite insect–plant–pathogen interactions.

## Supplementary Material

Supplementary material

## Data accessibility

The data cited in this research are available from the Short Read Archive of NCBI accession no. SRP149658.
