# Supplementary material for: Armet, an aphid effector protein, induces pathogen resistance in plants by promoting the accumulation of salicylic acid
Source: Philos Trans R Soc Lond B Biol Sci. 2019 Jan 14;374(1767):20180314. doi: 10.1098/rstb.2018.0314 (PMC6367143; doi:10.1098/rstb.2018.0314)
Supplement: Supplementary material [file rstb20180314supp1.pdf]

Electronic supplementary material

Armet, an aphid effector protein, induces pathogen resistance in plants by promoting the accumulation of salicylic acid

Na Cui[1,3], Hong Lu[1], Tianzuo Wang[2], Wenhao Zhang[2], Le Kang*[1], Feng Cui*[1]

[1]State Key Laboratory of Integrated Management of Pest Insects and Rodents, Institute of Zoology, Chinese Academy of Sciences, Beijing 100101, China

[2]State Key Laboratory of Vegetation and Environmental Change, Institute of Botany, Chinese Academy of Sciences, Beijing 100093, China

[3]University of Chinese Academy of Sciences, Beijing 100049, China

[*]Authors for correspondence:

Feng Cui

Tel: +86-10-64807218, Email: cuif@ioz.ac.cn.

Le Kang

Tel: +86-10-64807219, Email: lkang@ioz.ac.cn.

Materials and Methods

RNA extraction and real-time quantitative PCR

Total RNA was isolated from *N. benthamiana* and *M. truncatula* leaves using the RNeasy Plant Mini Kit (Qiagen, Hilden, Germany) according to the manufacturer's instructions. Six biological replicates were prepared; one replicate contained four *N. benthamiana* leaves or three *M. truncatula* leaves. Total RNA was isolated from *M. persicae* and *Locusta migratoria* using TRIzol reagent (Invitrogen, Carlsbad, CA, USA). RNA was treated using the TURBO DNA-free kit (Ambion, Austin, TX, USA) to remove genomic DNA contamination. One microgram of total RNA was reverse-transcribed to cDNA using the Superscript III First-Strand Synthesis System (Invitrogen) and oligo-dT primers (Promega, Madison, WI, USA).

Real-time quantitative PCR (qPCR) was used to quantify the transcript levels of *PDF1.2* (KP017278 in GenBank), *SABP2* (JX317629), *SAMT* (JX317630), *ICS* (ABX55938), *EDS1* (AAL85347), *SAGT* (Niben101Scf00788g02015 in the genome), *PR1* (Niben101Scf03376g03004), *BGL2* (Niben101Scf04869g03002), *CHS* (Niben101Scf00536g15017), and *PAD4* (Niben101Scf09577g01001) in *N. benthamiana* using the corresponding primers. The transcript levels of 18 genes in the plant-pathogen interaction pathway were quantified as previously described [18]. The transcript levels of *ApArmet* in transgenic *N. benthamiana* and *M. truncatula* were quantified using the primer pair *ApArmet*-q-F/*ApArmet*-q-R. The transcript levels of elongation factor-1 alpha (*ef1α*) of *N. benthamiana* (XM_019399807 in GenBank) and *actin* of *M. truncatula* (XM_003621971) were quantified using the primer pairs *ef1α*-q-F/*ef1α*-q-R and *actin*-q-F/*actin*-q-R, respectively. The primer sequences are listed in electronic supplementary material, Table S1. qPCR was performed in a Light Cycler 480 II instrument (Roche, Basel, Switzerland). The thermal cycling conditions were as follows: 95 °C for 2 min followed by 40 cycles of 95 °C for 30 s, 60 °C for 30 s, and 68 °C for 40 s. The transcript level of each gene relative to that of *ef1α* or *actin* was calculated and is presented as the mean ± standard error (SE). Differences were analyzed using one-way ANOVA for multiple comparisons or the *t*-test for pairwise

comparisons in SPSS 17.0.

Protein expression and purification

The cloning, expression, and purification of ApArmet have been described previously [1]. The open reading frames (ORFs) of Armets from *M. persicae* (MpArmet, XM_022311732 in GenBank) and *L. migratoria* (LmArmet, MH507503 in GenBank) were cloned using the primer pairs MpArmet-F/MpArmet-R and LmArmet-F/LmArmet-R, respectively (electronic supplementary material, Table S1). MpArmet and LmArmet without signal peptide-encoding sequences were constructed in the pET28a vector using the primer pairs MpArmet-NcoI-F/MpArmet-XhoI-R and LmArmet-NcoI-F/LmArmet-XhoI-R, respectively (electronic supplementary material, Table S1). The protocols used for expression and purification of MpArmet and LmArmet were the same as those used for ApArmet.

Transcriptomic sequencing and analysis

Total RNAs were sent to the BGI Company (Shenzhen, China) for RNA-seq analysis using the single-end digital gene expression sequencing strategy. *ApArmet* transgenic *N. benthamiana* was sequenced on an Illumina HiSeq 2000 sequencer (Illumina, Inc., San Diego, CA, USA). *N. benthamiana* and *M. truncatula* infiltrated with ApArmet and *ApArmet* transgenic *M. truncatula* were sequenced on a BGISEQ-500 sequencer. Four leaves from *N. benthamiana* and three leaves from *M. truncatula* were collected for RNA extraction as a biological replicate. Three biological replicates were prepared for ApArmet-infiltrated *N. benthamiana* and *M. truncatula* and *ApArmet* transgenic *N. benthamiana*, and two biological replicates were prepared for *ApArmet* transgenic *M. truncatula*. At least 12 million clean reads were obtained for each sample and mapped to the *N. benthamiana* genome (http://bti.cornell.edu/our-research/enabling-technologies/nicotiana-benthamiana/) and the *M. truncatula* genome (http://datacommons.cyverse.org/browse/iplant/home/mtruncatula/public/Mt4.0/Annotation/Mt4.0v2) using HISAT [2] and Bowtie2 [3]. Reads of each sample were deposited in the Short Read Archive of the National Center for Biotechnology Information (NCBI) under the accession number SRP149658. Gene expression levels were evaluated by RSEM [4], and differentially expressed genes were analyzed using

the Noiseq package [5] with a fold change threshold ≥ 2 and divergence probability ≥ 0.8. Differentially expressed genes were functionally annotated using the Kyoto Encyclopedia of Genes and Genomes (KEGG) (http://www.kegg.jp/).

[1] Wang W, Dai HE, Zhang Y, Chandrasekar R, Luo L, Hiromasa Y, Sheng CZ, Peng GX, Chen SL, Tomich JM, et al. 2015 Armet is an effector protein mediating aphid-plant interactions. *FASEB J*. **29**, 2032-2045. (doi:10.1096/fj.14-266023)

[2] Kim D, Landmead B, Salzberg SL. 2015 HISAT: a fast spliced aligner with low memory requirements. *Nat. Methods* **12**, 357-U121. (doi:10.1038/Nmeth.3317)

[3] Langmead B, Trapnell C, Pop M, Salzberg SL. 2009 Ultrafast and memory-efficient alignment of short DNA sequences to the human genome. *Genome Biol*. **10**, R25. (doi:10.1186/gb-2009-10-3-r25)

[4] Li B, Dewey CN. 2011 RSEM: accurate transcript quantification from RNA-Seq data with or without a reference genome. *BMC Bioinformatics* **12**, 323. (doi:10.1186/1471-2105-12-323)

[5] Tarazona S, Garcia-Alcalde F, Dopazo J, Ferrer A, Conesa A. 2011 Differential expression in RNA-seq: A matter of depth. *Genome Res*. **21**, 2213-2223. (doi:10.1101/gr.124321.111)

Figure S1. Jasmonic acid (JA) concentrations in ApArmet protein-infiltrated (A) and control (B) *Nicotiana benthamiana* measured using ultra-performance liquid chromatography-tandem mass spectrometry. Leaves infiltrated with purified product from the pET28a empty vector were used as a control. The JA concentration was measured at 60 h post-infiltration.

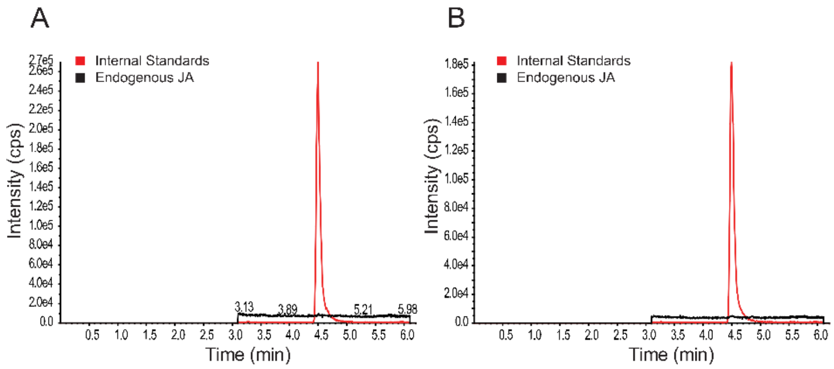

Figure S2. Armet enhances plant resistance to bacterial pathogens but does not enhance resistance to aphids. (A) and (B) Survival curve and reproductive rate of *Myzus persicae* on ApArmet protein-infiltrated *Nicotiana benthamiana*. (C) and (D) Survival curve and reproductive rate of *Acyrthosiphon pisum* on ApArmet protein-infiltrated *Medicago truncatula*. Leaves infiltrated with the purified product obtained from cells infected with the pET28a empty vector were used as a control. n.s., no significant difference. (E) and (F) Number of *Pseudomonas syringae* pv. *tabaci* colonies from ApArmet protein-infiltrated *N. benthamiana* leaves at 6 and 9 d post-infiltration (dpi) of bacteria. Leaves infiltrated with the purified product from the pET28a empty vector were used as a control. *, $P < 0.05$; ** $P < 0.01$.

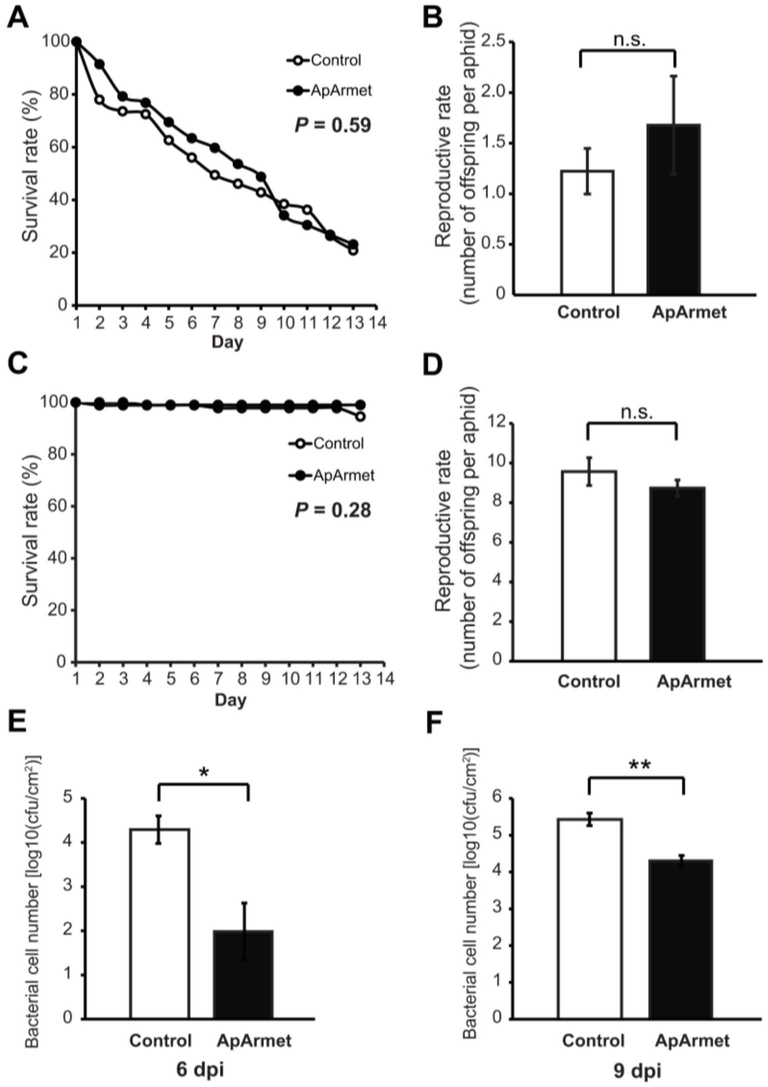

Figure S3. Phylogenetic analysis and amino acid sequence alignment of insect Armets. (A) Unrooted phylogenetic tree of 133 insect Armets constructed using the neighbor-joining method (p-distance and pairwise deletion) in MEGA 6.0. Bootstrap analysis of 1000 replicates was applied to evaluate the confidence of the tree topology. Aphid Armets are shown in red. (B) Amino acid sequence alignment of seven aphid Armets and other insect Armets. The threshold for shading is 43% identity.

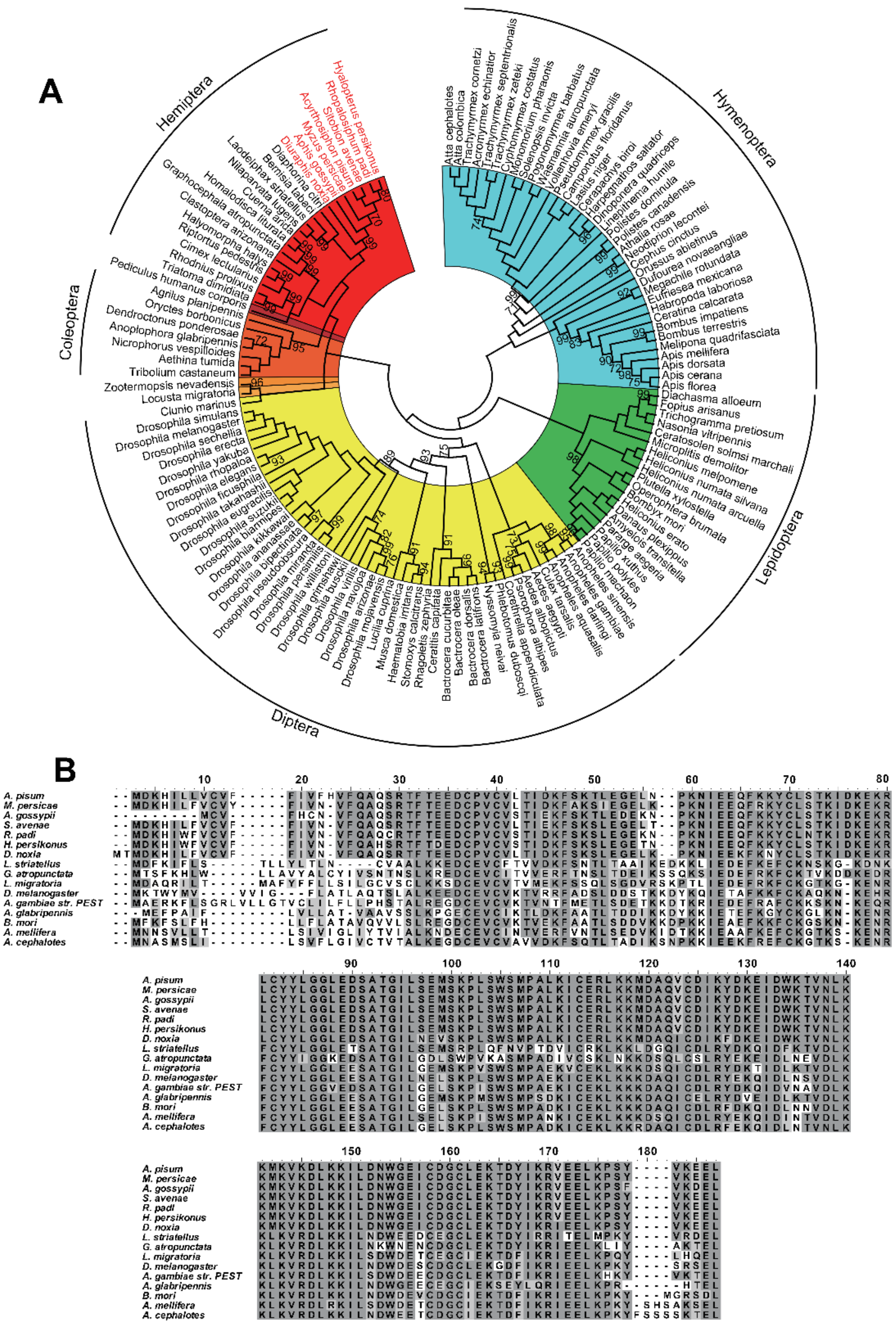

Table S1. Primers used in this study.

Primer name	Primer sequence
NbEf-1-F	CACCATTGATATTGCCTTGT
NbEf-1-R	CTGTCCATCCTTAGAGATACC
MtActin-F	ACGAGCGTTTCAGATG
MtActin-R	ACCTCCGATCCAGACA
ApArmet-F	CACCATGCAATCGCGAACATTTAC
ApArmet-R	TTATAACTCTTCCTTGACGTAAG
ApArmet-KpnI-F	CGGGTACCATGCAATCGCGAACATTTAC
ApArmet-SpeI-R	GGACTAGTCCTTATAACTCTTCCTTGACGTAAG
ApArmet-qPCR-F	GTTACTATTTGGGCGGATTA
ApArmet-qPCR-R	TATCGCAGACTTGAGCAT
SABP2-F	CACCATGAAGGAAGGAAAACACTTTG
SABP2-R	TCAGTTGTATTTATGGGCAATC
SABP2-qPCR-F	ATGCCATGGAGGTTGGAGTT
SABP2-qPCR-R	ACCAAGACTATGCCCCACCA
SABP2-I miR-s	GATAACATAGATGTCAGTCGCTCTCTCTCTTTTGTATTCC
SABP2-II miR-a	GAGAGCGACTGACATCTATGTTATCAAAGAGAATCAATGA
SABP2-III miR*s	GAGAACGACTGACATGTATGTTTTCACAGGTCGTGATATG
SABP2-IV miR*a	GAAAACATACATGTCAGTCGTTCTCTACATATATATTCCT
SABP2-A	CACCCTGCAAGGCGATTAAGTTGGGTAAC
SABP2-B	GCGGATAACAATTTCACACAGGAAACAG
SAMT-F	CACCATGAAGGTTGTTGAAGTTCT
SAMT-R	TTAATTTGTTTTGGTCAATGAG
SAMT-qPCR-F	CGCAGTTGAGAAGGAGGGTT
SAMT-qPCR-R	TGTCTTCTCTTTAGACATGCGG
MpArmet-F	ATGGATAAACACATATTG
MpArmet-R	TTATAACTCTTCCTTGAC
MpArmet-NcoI-F	GTACCCATGGGCATGCGAACATTTACTGAAGAAG
MpArmet-XhoI-R	GGGCCTCGAGTAACTCTTCCTTGACGTAAG
LmArmet-F	ATGGATGCGCAAAGGATTC
LmArmet-R	TTAAAGTTCCTGGTGGAGATAC
LmArmet- NcoI-F	GTACCCATGGGCATGCTGAAGAAATCTGATTGTG
LmArmet- XhoI-R	CCGCTCGAGAAGTTCCTGGTGGAGATAC
LMArmet-5’race-1	GCGACTGGGATGAGACTTGTGAAGGC
LMArmet-5’race-2	GGTCTATGCCAGCTGAGAAAGTGTGTGA
SAGT-qPCR-F	ACTCCTGTGTCAATCGAGGC
SAGT-qPCR-R	TTCCCACTTCAACAGCCCAA
ICS-qPCR-F	TAGCTGCCAGATTTAGTTCCTCA
ICS-qPCR-R	TGGTTTTGGAGTCCGGAGAA
CHS-qPCR-F	GCAGCCCAAACTTTATTGCC

CHS-qPCR-R	GCCCGAAGTTTCTCTGGCT
EDS1-qPCR-F	TCCGAAACAGAGGAAAGCTTAGA
EDS1-qPCR-R	CTGCTGCACGAAGACACAAC
PAD4-qPCR-F	TTCGTACAAGCAACGAGGCA
PAD4-qPCR-R	CATTTACCCACTTCGCACGC
BGL2-qPCR-F	CGAATACCTACCCACCGGAAA
BGL2-qPCR-R	TGCGACATTAACAGTGTTGGAA
PR1-qPCR-F	GCAGTTGTAGGCGTAGAA
PR1-qPCR-R	CCAACACGAACCGAGTTA
PDF1.2-qPCR-F	CAGAGATGGGACCAACGACA
PDF1.2-qPCR-R	CTTCGGTCAAACAGACGGTG
